# Study protocol: glycaemic outcomes in people with type 2 diabetes initiating continuous glucose monitoring: the 2GO-CGM study

**DOI:** 10.1007/s40200-023-01244-y

**Published:** 2023-06-15

**Authors:** Claire S. Lever, Jonathan A. Williman, Alisa Boucsein, Antony Watson, Rachael S. Sampson, Oscar T. Sergel-Stringer, Celeste Keesing, Lynne Chepulis, Benjamin J. Wheeler, Martin I. de Bock, Ryan G. Paul

**Affiliations:** 1 Te Huataki Waiora, School of Health, University of Waikato, Hamilton, Aotearoa New Zealand; 2 Waikato Regional Diabetes Service, Te Whatu Ora Health New Zealand Waikato, Hamilton, New Zealand; 3 Aotearoa Diabetes Collective, Hamilton, Waikato New Zealand; 4 Department of Population Health, University of Otago, Christchurch, New Zealand; 5 Department of Women’s and Children’s Health, Dunedin School of Medicine, University of Otago, Dunedin, New Zealand; 6 Department of Paediatrics, University of Otago, Christchurch, New Zealand; 7 Pinnacle Midlands Health Network, New Plymouth, New Zealand; 8 Department of Paediatrics, Te Whatu Ora Southern, Dunedin, New Zealand; 9 Department of Paediatrics, Te Whatu Ora Health New Zealand Waitaha Canterbury, Christchurch, New Zealand

**Keywords:** Type 2 diabetes, Continuous glucose monitoring, Health economics, Quality of life

## Abstract

**Purpose:**

Improving glycaemic control in type 2 diabetes (T2D) is essential to reducing social and health-economic burden of diabetes-related complications. Continuous glucose monitoring (CGM) has been established as beneficial in improving glycaemic control and reducing hypoglycaemia in people with type 1 diabetes, however data in T2D is limited. This study has been designed to assess the effect of initiating real-time CGM (rtCGM) on glycaemic control in a high-risk population of adults with T2D. Secondary objectives are to assess the cost-effectiveness and safety of rtCGM, and the effects of rtCGM on diet/lifestyle and the burden of diabetic complications, including cardiovascular risk.

**Methods:**

This multicentre randomised controlled trial (RCT) will be conducted at three sites in New Zealand (Waikato, Christchurch and Dunedin). Eighty adults with T2D on insulin with suboptimal glycaemic control (HbA1c > 8.0% or 64 mmol/mol) will be randomised 1:1 to rtCGM or routine care with self-monitoring of blood glucose levels (SMBG) for three months. This intervention phase will be followed by a three-month continuation phase where SMBG group crossover to use rtCGM. Participants will then be invited to join the extension phase with continued use of rtCGM for a further 12 months. During the extension phase, both groups will independently titrate their insulin under the remote supervision of prescribing diabetes nurse specialists following an insulin titration algorithm. The primary outcome of the study is time in target glucose range (3.9–10 mmol/L or 70–180 mg/dL; TIR). Secondary outcomes include CGM metrics as per consensus statement recommendations, and HbA1c. Additional planned analyses include cardiovascular risk profile, incremental cost-effectiveness analyses, dietary patterns, and qualitative analyses.

**Trial registration number:**

The trial was registered with the Australian New Zealand Clinical Trials Registry (ACTRN12621000889853) on 8 July 2021 and the World Health Organisation International Clinical Trial Registry Platform (Universal Trial Number U1111–1264-5822).

## Background

Treating to glycaemic target is well established as imperative to reducing diabetes-related complications [1]. However, achieving and maintaining glycaemic control in T2D remains a challenge. Despite developments in glucose lowering therapies and evolving clinical guidance in T2D management, at least 50 % of people with T2D are not achieving glycaemic targets [2–4]. Moreover, indigenous populations are less likely to achieve glycaemic targets, and experience a higher incidence and earlier onset of complications and mortality when compared to their non-indigenous counterparts [5–8]. The existence of these trends in the context of increasing health expenditure commands new approaches to improving glycaemic outcomes.

While reasons for poor glycaemic control are complex and multifactorial [9, 10], a paucity of self-monitoring of blood glucose is a significant barrier to improved diabetes management [10–12]. Self-monitoring of blood glucose (SMBG) remains an integral component of glycemic management for people using insulin [13]. Unfortunately, adherence to SMBG is often low due to the discomfort of finger pricks and the intrusive nature of the testing regimen to daily life [10, 11]. Without sufficient glucose monitoring, self-management and supported management of hyperglycaemia is impeded [14–16]. These barriers are significantly reduced in type 1 diabetes (T1D) with the use of CGM [17] and as such, CGM may offer a solution to delays in intensification of glycaemic management in T2D.

Real-time CGM (rtCGM) measures interstitial glucose levels and automatically sends glucose values via Bluetooth to a receiving device (e.g., smartphone or insulin pump), usually every 5 minutes. The CGM sensors are inserted by the user every 7–14 days (device dependant). The device also reports on glucose trends and rate of change and allows the user to set and respond to individualised alerts for low, predicted-low, high and rapidly changing glucose levels.

Real-time CGM has been shown to improve glucose control and reduce hypoglycaemia in T1D [17–22] and has become a standard of care [13, 23, 24]. Older generation rtCGM has largely demonstrated favourable outcomes in adults with T2D on insulin who are not reaching their glycaemic targets [25–29], but high-quality data of CGM use in T2D is limited. Further, there is a lack of contemporary data using modern rtCGM, which is more accurate and does not require finger prick glucose calibration. Therefore, modern rtCGM has a greater potential to be utilised by people with T2D to improve glycaemic outcomes.

Therefore, we aim to study the impact of rtCGM on a population of people with T2D at high risk of developing complications using a variety of outcome measures including glycaemic metrics, cardiovascular risk outcomes (e.g. blood pressure, HsCRP, lipids, urinary albumin:creatinine ratio), quality of life, and cost effectiveness.

## Methods

### Study design

This multi-centre, randomised controlled trial (RCT) of 80 participants will be conducted in Aotearoa New Zealand over a period of 14 weeks, including a 2-week run-in period for blinded CGM data to be collected from all participants. Following the 2-week run-in period, participants will be randomised 1:1 to either the rtCGM intervention arm (Dexcom G6 rtCGM system (Dexcom Inc., San Diego, CA)) or the control arm (SMBG) with a CareSens premier meter (i-SENS Inc., Seoul, Korea)) for a period of 12 weeks. The primary endpoint will be measured in the final two weeks of the RCT phase (week 10–12), with blinded rtCGM data collected from the control group and compared with unblinded rtCGM data from the intervention group. The 12-week RCT phase will be followed by a continuation phase, where those initially randomised to routine care will cross over into the rtCGM intervention for a further 12 weeks. Following this, participants will be invited to join the extension phase of this study and continue rtCGM use for a further 12 months. As such, the expected duration of subject participation is 78 weeks. At least five visits to the study centre and six ‘remote reviews’ are planned for participants over the study duration (see Table 1). During the 12-month extension phase participants will switch from using the Dexcom G6 system to the Dexcom G7 system, once available.

**Table 1:** 2GO-CGM study schedule of assessments

	Baseline	Run-in period 14 days	Treatment period week 1–12				Continuation PERIOD Week 12–24			EXTENSION PERIOD Week 25–77			
	Clinic visit		Clinic visit day 1	Remote review week 2, 8	Week 10–12	Clinic visit end of study week 12	Clinic visit week 12	Remote review week 14, 20	Clinic visit end of study week 24	Week 38 remote review	Clinic visit week 51	Week 64 remote review	Clinic visit end of extension study week 77
Informed consent	X								X				
Demographics	X												
HbA1c Height, weight, BMI	X		X			X	X		X		X		X
Medical review	X					X	X		X		X		X
Blood pressure	X					X	X		X		X		X
Urine pregnancy test	X												
Fasting lipids, FBC, glucose, LFTS, eGFR	X					X	X		X		X		X
Urine ACR	X					X	X		X		X		X
Blinded CGM (14 days)		X			X (control arm)								
Return to clinic after 7 days of blinded CGM for new sensor		X			X (control arm)								
CGM training			X				X						
Electronic review				X				X		X		X	
AE collection		X	X	X		X	X	X	X	X	X	X	X
Sleep quality		X				X							
Food diary		X				X					X		
Qualitative interview						X (CGM arm)			X (control arm)			X (if used both G6 and G7 CGM)	
Retinal scan results	X												

The study protocol was approved by the New Zealand Health and Disability Ethics Committee prior to study commencement (21/CEN/75).

### Recruitment and eligibility

The 2GO-CGM trial will enrol 80 participants, aged 16 years and older with T2D. Participants will be recruited from Waikato, Canterbury and Southern regions, collectively servicing approximately 65,000 people with T2D [30]. To be eligible, participants must have a sub-optimal Hba1c (HbA1c > 8.0% or 64 mmol/mol) despite receiving at least 0.2 units of insulin per kilogram, per day, for at least 3 months prior to study enrolment. Full eligibility criteria are presented in Table 2. While not an eligibility criterion, it is expected that most participants will be rtCGM naïve, given that rtCGM is currently unfunded and therefore not widely used by people with T2D in New Zealand.

**Table 2:** Eligibility criteria for participation in the 2GOCGM study

Inclusion criteria	Exclusion criteria
• Type 2 diabetes as per American Diabetes Association classification • HbA1c >8.0% or 64 mmol/mol • Minimum daily insulin use of ≥0.2 units of insulin/kg/day for >3 months • Aged 16 years and over • Be willing and able to conform to the study protocol • Have access to a smartphone or computer with internet connection	• History of type 1 diabetes • History of other types of diabetes such as diabetes secondary to pancreatitis • Has had a hospital admission for hyperglycaemia in last 6 months • Use of systemic corticosteroids >14 days, or repeated pharmacologic systemic courses of corticosteroids • Recurrent or chronic systemic infections that, in the view of the investigator, would significantly impact on glycaemia. • Major cardiovascular event or major surgery in last 3 months • Active malignancy requiring ongoing treatment • Previous or planned bariatric surgery • Pregnancy, or plan to become pregnant while participating in study • Any other reason that investigator feels may not be in best interest of patient to participate

Māori are the tāngata whenua (indigenous people) of New Zealand and are disproportionately affected by T2D. Māori are an underrepresented population in both CGM use and research, and recruitment of Māori people will be prioritised for the study group to be representative of the T2D population in New Zealand.

Potential study candidates will be identified by their local healthcare professionals (e.g. general practitioners, practice nurses and specialist diabetes clinicians) and agree to be phoned by a study team member for more information about the study and to confirm eligibility. Māori and Pacific participants may prefer an initial face to face appointment for *whakawhānaungatanga* (making a connection with research team member/s) before discussing the study and eligibility [31] and will be offered this opportunity. Potential participants will be given adequate time to review the patient information and consent form (PCIF) before indicating whether they would like to join the study. Processes of obtaining formal consent will meet the requirements of ISO 14155:2011 and Good Clinical Practice. Participants will confirm consent at the first screening appointment (Day 0) where they will provide written informed consent before any study specific assessments are performed. Additional consent will be sought for participation in interviews regarding the use of the Dexcom G6/G7 system. Table 3 shows the baseline information that will be collected once consent has been obtained and the participant has been screened, confirmed as eligible and enrolled into the study.

**Table 3:** Baseline assessments for 2GO-CGM study

Demographic	Date of birth Ethnicity Gender
Anthropometric	Height Weight Body Mass Index (BMI) Seated blood pressure, recorded as average of 2 measurements at least 5 minutes apart
Diabetes	Date of diagnosis HbA1c (local laboratory value taken within the last 30 days) Number of episodes of severe hypoglycaemia, defined as coma or convulsion requiring assistance from others, in the 12 months prior to baseline visit. Number of hospitalisations in the last 12 months (diabetes and non-diabetes related)
Clinical	Medical history, including co-morbidities Medication list Known allergies Results of retinal screening within past 24 months (if not done, retinal photography will be arranged) Insulin injection sites checked for lipohypertrophy Check of insulin management and injection techniques
Lifestyle	Smoking status, alcohol, recreational drug use
Laboratory	Fasting glucose Fasting lipid profile Full Blood Count Liver function tests (AST and ALT) Estimated glomerular filtration rate High-sensitivity C-reactive protein Urine albumin creatinine ratio

### Sample size and power calculation

In a recent study examining intermittent CGM in a population with type 2 diabetes, baseline time in range (TIR) was 62.5% ± 20% [32]. Our inclusion criteria predicts a population that are likely to have lower time in range, which we estimate at 56% ± 20%. To detect a difference to a mean improvement of 14% TIR (to the target TIR of 70% set by international guidelines), at 80% power and α of 0.05, we would need 33 participants in each arm. The sample size has been inflated to 40 participants in each arm to account for possible loss-to-follow-up. This power calculation is a conservative estimate, and we are likely to be able to detect a smaller difference. For example, adjusted for baseline TIR a sample size of 40 per group will give 78% power to detect a difference of 10% TIR, assuming a within-person standard deviation of 18.5% (correlation coefficient of r = 0.58) [32], and that there is no loss-to-follow-up. The mean between-group difference in TIR at 12 weeks will be estimated with 95% confidence intervals using analysis of covariance, a general linear model that adjusts for TIR at baseline.

### Randomisation

The 2GO-CGM participants will be randomly allocated to use Dexcom G6 CGM or routine care with CareSens Premier meter with an allocation ratio of 1:1. The study statistician will prepare a computer-generated randomisation list with permutated blocks of random size.

The randomisation list will be stratified by:

1. Use of metformin (Yes/No)
2. Use of SGLTi or GLP agonist (Yes/No)

Use of this stratification plan is based on recent public funding for SGLTi and GLP agonists as second-line agents in New Zealand. Given this recent change these agents have not been widely used and therefore participants will be randomised based on prior use of either agent on entry to the study.

The randomisation list will be loaded into the REDCap (Research Data Capture) database by the study statistician immediately prior to moving the project to production status. Once the project is moved to production, the randomisation list is locked and becomes unmodifiable and inaccessible to the study team, thereby preserving allocation concealment. The study statistician will not be involved in participant enrolment. Patients who give consent for participation, meet the eligibility criteria, and are enrolled in the study, will be given a unique study identifier. After stratification variables have been entered into REDCap, research staff with authorisation to randomise participants may click the ‘randomise’ button, which will assign the treatment to the study number according to the next allocation on the randomisation list. This process will lock the fields containing the treatment group and stratification variables. Blinding will not be possible due to the nature of the technology.

## Baseline measures

### Demographics

Demographic data (age, gender and ethnicity) will be collected from participants. Home addresses will be used to assess socioeconomic deprivation scores using NZDep2018. NZdep2018 is an area-based measure of deprivation, based on New Zealand census data [33]. Deprivation deciles between one and 10 are allocated to areas of approximately 100–200 people: decile one representing areas with the lowest deprivation scores and decile 10 representing areas of highest deprivation.

### Anthropometric

Investigative staff members will collect anthropometric measurements (height and weight) using calibrated instruments and standard clinical procedures. Weight will be measured to the nearest 0.1 kg, with shoes and heavy clothing removed. Height will be measured to the nearest 0.5 cm. Height and weight will be used to measure body mass index (BMI) using standardised formula.

### Clinical characteristics

Blood pressure will be measured in seated position with an appropriately sized cuff. Two blood pressure measurements will be taken at least 5 minutes apart at each review point and the average of both measures recorded.

Retinal screening results completed in the last 2 years will be recorded, or the participant will be referred for screening if no result is available. Medical history including diabetes-related complications and a complete list of medications will be recorded at baseline.

### Blinded rtCGM

All participants will have at least 10 days of glucose data recorded by blinded Dexcom G6 device in the 2-week run-in phase of the trial. This data will be used by prescribing diabetes nurse specialists (DNS) to maximise non-insulin glucose lowering medications for all participants prior to randomisation into study group.

## Study groups

### Intervention arm (Dexcom G6 system)

At the beginning of the RCT phase, participants randomised to the intervention arm will receive training on the Dexcom G6 system (Dexcom Inc., San Diego, CA) by investigative staff. Training will include: application and activation of the G6 system, setting and responding to alarms/alerts, interpreting arrows and trend graphs, using the share/follow function (if applicable) and calibration (if indicated).

Participants will have the Dexcom G6 app downloaded onto their compatible smartphone or will use a Dexcom receiver. Participants will also use Dexcom Clarity; glucose depository software which allows participants and investigative staff to view glucose data, and patterns, trends, and statistics. Participants will have individual Dexcom Clarity accounts, identifiable only by their study ID. Data from these accounts will be shared with the study team Dexcom Clarity Professional account, allowing for remote review of participant data by investigative staff.

For participants using Dexcom G6 app on their smartphone, the Dexcom Clarity app will also be downloaded onto their phone and data will automatically upload to Dexcom clarity when connected to the internet. Participants using the Dexcom receiver will upload their glucose data manually to the Dexcom Clarity website at least once every 30 days, and prior to review points.

### Control arm (SMBG)

Funded glucose monitoring in New Zealand consists of a CareSens glucometer (i-SENS Inc., Seoul, Korea) and test strips. Participants randomised to the control arm will use a Caresens Premier meter, due to the Bluetooth functionality of the device. Participants will have the SmartLog app downloaded onto their compatible smartphone or have SmartLog software set up on their computer. SmartLog is diabetes management software that allows users to view, analyse and share blood glucose data. For participants using SmartLog on their phones, glucose data will be transmitted via Bluetooth when the meter is on and in close proximity to the smartphone. Participants who are unable to use SmartLog on their phones will download data manually to SmartLog on their computer as required. Participants in the control arm will share their glucose data with study staff prior to review points.

Participants in the control arm will receive training on the Caresens Premier Meter and SmartLog software. Training will include: revision of glucose testing technique and glucometer use, pairing the glucometer with SmartLog app on phone, or downloading meter results to SmartLog software, understanding error messages, interpreting glucose trends and graphs on SmartLog and exporting data from SmartLog to the investigative staff. At the end of the RCT phase participants in the control arm will receive the same training on the Dexcom G6 system as the intervention group and use the Dexcom G6 for the continuation phase of the trial (week 12 to 24).

## Glycaemic and cardiovascular risk management

Participants from all localities will be supported by prescribing DNS based at the Waikato study site, with endocrinologist support if required. For all participants, regardless of randomisation, the prescribing DNS will assist participants to manage dietary and lifestyle factors as well as medications, with a focus on participant self-adjustment of insulin during the RCT and continuation phases. Prescribing DNS will follow an insulin titration algorithm (Appendix) to ensure participants receive standardised insulin adjustment advice based on available glucose data. The prescribing DNS will make medication, diet and lifestyle recommendations (as required) at all clinic visits and remote reviews. Medication recommendations will occur as follows:

### Run-in phase

During the run-in phase prescribing DNS will adjust non-insulin glucose lowering medications with the aim to maximise these medications prior to the RCT phase for all study participants. DNS will also assess cardiovascular risk factors and adjust anti-hypertensive and lipid lowering medications in the run-in phase and continuation phase, based on national guidelines [34].

### Randomised controlled trial phase

The RCT phase will be preserved for insulin changes only, unless medication adjustments by DNS are required in this phase for participant safety. For non-urgent cardiovascular risk management during the RCT phase, DNS will request follow up by the participant’s usual care team. Prescribing DNS will provide participants with written insulin titration instructions to follow between scheduled study reviews.

### Continuation and extension phase

During the continuation and extension phases, prescribing DNS will adjust all glucose lowering and cardiovascular medications if required and provide participants with written insulin titration instructions to follow between scheduled study reviews.

## Outcome measures

Study endpoints will follow current international consensus guidelines [35] and include primary and secondary (including binary and composite) endpoints.

### Primary endpoint

The primary objective of this study is to compare the percentage of TIR (3.9–10.0 mmol/L; 70–180 mg/dL) between the intervention and control groups during the last two weeks of the RCT phase. Participants in the control arm will have this collected by blinded CGM during the last two weeks of the RCT phase only.

### Secondary endpoints

#### Secondary efficacy endpoints

The following measures of glycaemic control collected during the last 14 days of the RCT phase (~weeks 10 to 12) will be tested following the primary endpoint in a hierarchical fashion in the order listed below.

1. % CGM time > 10.0 mmol/L (180 mg/dL)
2. % CGM time > 13.9 mmol/L (250 mg/dL)
3. Mean sensor glucose (mmol/L)
4. HbA1c measured at 12 weeks via local laboratory testing
5. % CGM time < 3.9 mmol/L (70 mg/dL)
6. % CGM time < 3.0 mmol/L (54 mg/dL)

#### Secondary exploratory endpoints

All other measures are considered exploratory and will use the false discovery rate to control Type 1 error.

- % CGM time 3.9–7.8 mmol/L (70–140 mg/dL)
- Glycaemic outcomes differentiated as 24 hours, day (0600–2359 hours) and night (0000–0559 hours)
- Glucose variability (expressed as primarily a coefficient of variation and secondly a standard deviation).

#### Binary outcomes

Proportion of participants with:

- TIR >70% of each day
- TIR with ≥5% points improvement from baseline
- TIR ≥10% points improvement from baseline
- Time < 3·9 mmol/L (70 mg/dL) for <4% of each day
- Time < 3·0 mmol/L (54 mg/dL) for <1% of each day
- Time > 10.0 mmol/L (180 mg/dL) for <25% of each day
- Time > 13.9 mmol/L (250 mg/dL) for <5% of each day

#### Composite endpoints

Proportion of participants with:

- Improvement in HbA1c of >0·5% points without an in increase in time < 3.0 mmol/L (< 54 mg/dL)
- More than 10% points improvement in percentage of TIR without an increase in time < 3.0 mmol/L (54 mg/dL) of >0.5%
- Mean glucose <8.6 mmol/L (154 mg/dL) and < 1% time < 3.0 mmol/L (<54 mg/dL)
- More than 70% TIR and < 4% time < 3.9 mmol/L (70 mg/dL)
- More than 70% TIR and < 1% time < 3.0 mmol/L (54 mg/dL)

#### Cardiovascular profile

The following biochemistry markers and anthropometric measurements will be collected at baseline, week 12 and week 24. Samples for biochemistry data will be collected and processed at local community laboratories.

- Fasting lipid profile
- Fasting glucose
- Urine micro-albuminuria
- Estimated glomerular filtration rate
- Full blood count
- Liver function tests
- High sensitivity C-reactive Protein
- Weight
- Body Mass Index
- Blood pressure

### Psychosocial Factors

#### Sleep quality

The Pittsburgh Sleep Quality Index (PSQI) measures self-reported sleep quality and quantity over the previous month via a 19-item questionnaire. Answers generate seven “component” scores of sleep: subjective sleep quality, sleep latency, sleep duration, habitual sleep efficiency, sleep disturbances, use of sleeping medication and day-time dysfunction [36]. The component scores are aggregated into a global score of 0–21 for adults, with scores higher than five indicating poor sleep. The two items related to sleeping with a bed partner are not included for adolescents.

#### Eating behaviours (4-day diet record)

Participants will be asked to complete a four-day diet record during the run-in phase, and again between week 10 and week 12 of the RCT phase and after 12 months of rtCGM sensor use. The diet record will be completed over three, non-consecutive weekdays and one weekend day on the Research food diary app (Xyris Software (Australia) Pty Ltd) on the participant’s phone. The Research food diary app allows participants to record all food and drinks consumed by searching the food database, scanning barcodes or taking photos. The participants’ diet records will be shared with the investigative staff who will view them in Easy Diet Diary Connect (Xyris Software (Australia) Pty Ltd) and transfer the data to FoodWorks (Xyris Software (Australia) Pty Ltd). Data will be analysed for changes in total energy, macronutrients, and food groups.

### Platform Performance

Platform performance will be gauged by reporting on technical issues with both Dexcom G6 and CareSens/SmartLog systems. Investigative staff will troubleshoot technical issues with participants through the study and record device deficiencies in REDcap. Average Dexcom G6 sensor lifespan information will also be collected throughout the study. Platform performance and usability will also be measured through qualitative interview-based assessment of participant experience using rtCGM (both G6 and G7).

### Health Economic Analysis

If a statistically significant effect on the primary outcome (TIR) is detected, a cost-effectiveness analysis will be undertaken. Cost per percentage point of TIR will be calculated, using incremental cost-effectiveness analysis (ICEA). Costs will be based on the total cost of the trial, excluding costs relating to evaluation of the trial. This conservative approach best approximates the cost of scaling up the treatment across a broader population. In addition, if suitable comparative studies can be found that enable conversion of TIR to Quality-Adjusted Life Years (QALY), a cost-utility analysis will be undertaken. This will calculate the cost-per-QALY-gained, using ICEA.

### Individual interviews

A purposive sample of participants who have used CGM for at least three months will be invited to participate in individual interviews, either face-to-face or via video conference. Written informed consent will be obtained before beginning interviews. The objective of the qualitative study is to explore the lived experience of participants who have used CGM. Indigenous (Māori) and non-indigenous interviews will be conducted separately to ensure rich data collection from Māori by using a Kaupapa Māori-informed framework [37]. This is a ‘by-Māori, for Māori’ approach to research with Māori that seeks to understand the research question within Māori ways of knowing and being [37]. For Māori, the interview and data analysis will be conducted by Māori research team members and will explore the participant experience of the model of care in 2GO-CGM study. Questions will focus on participant experiences of technology use, nurse prescribing and healthcare education, and the impact of these experiences on Te Ao Māori dimensions of health, including taha wairua (spiritual health), taha whānau (family health), taha hinengaro (emotional health), and taha tinana (physical health) [38]. For non-Māori, the line of questioning will be focused on the experience of wearing the CGM device (acceptability and usability), comparison to self-monitoring of blood glucose levels, and how the device modified participants’ health behaviours. The experience of all participants who have used both the Dexcom G6 and G7 system will also be explored. Data from Māori and non-Māori will be analysed separately via inductive thematic analysis [39].

### Safety/Adverse Events

Adverse events are any untoward medical events that occur during the study. The following adverse events (AEs) will be reported in this study:

1. Adverse device events (ADE): an AE related to the use of the study devices (e.g., CGM, glucometer or phone app).
2. Serious ADE (SADE), or serious AE (SAE): an AE that is fatal, or life-threatening, or causes permanent bodily impairment, or requires hospitalisation, or medical/surgical intervention to prevent serious sequelae.

AEs that do not meet SADE criteria will not be recorded (e.g., respiratory or gastro-intestinal illness). Furthermore, hyperglycaemia and hypoglycaemic events are a frequent anticipated event in insulin users and will not be recorded unless glycaemic excursions are severe and require assistance of a third party, including medical intervention (e.g., hypoglycaemia resulting in altered consciousness and hospitalisation). In these instances, events will be recorded as SAE. SAEs (particularly episodes of HHS, DKA and severe hypoglycaemia) will be notified to the primary investigator/s within 24 hours of the event. Device deficiencies, or technical inadequacies of a device’s quality, durability, reliability, safety or performance, will be assessed as per Good Clinical Practice guidelines (ISO 14155:2020) [40]. Each device deficiency will be assessed for SADE potential and reported to the coordinating principal investigator within one working day if SADE criteria is met. Participant user errors will not be recorded unless they result in a SAE, ADE or SADE. Adverse events will be collected continuously from study onset to study end. Data recorded will include start/stop date of the event, associated symptoms, treatment of event, seriousness, relationship to the investigational device(s), and outcome. All adverse events, regardless of the relationship of the event to the study devices, will be followed up until they have returned to baseline status, stabilised or until ongoing sequelae have been established.

### Data management

Electronic device data will be collected from the Dexcom CGM using the Dexcom Clarity software. During the study, study-specific source documents will be maintained in locked file cabinets in locked rooms, and/or password protected databases via password protected computers in locked rooms. Post-study, study-specific source documents will be archived at a secure site at the University of Otago. Source documents will be retained for at least 10 years, then destroyed. Identifiable data will be converted to a de-identified form at the study site, at which point it is entered into electronic case report forms using the secure data platform REDCap (Research Electronic Data Capture). The data platform complies with international and national regulatory requirements for electronic data capture systems in the countries where it is used. Data entry will be limited to designated study staff trained and experienced in transcribing data for this purpose. The de-identified database will remain on securely held University servers for up to approximately 15 years.

### Statistical analysis

Statistical analysis will be performed using up-to-date specialist software (R or Stata). Analysis will follow the intention-to-treat principle, with all participants who commence the RCT phase included in the data set as randomised. Two-sided P-values will be reported, with an alpha of 0.05 considered statistically significant. The type 1 error for primary and secondary efficacy outcomes will be controlled using an ordered testing procedure where formal testing stops as soon as a non-significant result (p > = 0.05) is observed. Analysis of secondary exploratory outcomes will use the two-stage Benjamini-Hochberg method to control the false discovery rate.

Real-time CGM data will be collected in the run-in phase and week 10–12 of the RCT phase and used for calculation of baseline and follow-up CGM metrics, respectively. Participant CGM metrics will only be set to missing if less than 24 hours of data has been collected. The primary endpoint (TIR 3.9–10.0 mmol/L; 70- 180 mg/dL) will be calculated for each participant by dividing the number of CGM measures in target range, by total number of CGM values recorded. The overall treatment effect (primary outcome) will be determined by fitting an ordinary least squares linear regression model with percentage time in target glucose range at 12 weeks as the dependent variable and treatment group as the independent variable. The model will adjust for stratification variables (baseline use of metformin and use of SGLTi or GLP agonist) and percentage time in target glucose range during the last two weeks of run-in. This model will be used to calculate point estimate, 95% profile likelihood confidence intervals, and p values for the treatment group difference at follow-up. Continuous secondary endpoints (e.g CGM metrics, anthropometric, clinical and laboratory cardiovascular measures and PSQI) will follow the same approach as described for the primary efficacy endpoint.

Real-time CGM data will be collected daily during the entire continuation and extension study phases. This data will be pooled with data collected during the run-in and RCT phases and analyses undertaken using linear mixed-effects models. These models combine observed between-group and within-person treatment effects, and allow for changes over time to be estimated. Model fixed effects will include current treatment (Dexcom CGM or SMBG), time on study and time on intervention. Individual variation in glycaemic control at baseline and changes over time will be allowed for using random effects.

## Discussion

Reducing the burden of T2D requires novel approaches to improving glycaemic management, particularly for indigenous populations who continue to experience suboptimal glycaemic control and inequities in diabetes-related outcomes. Real-time CGM may offer advances in glycaemic management in T2D without increasing the burden of disease. However, a lack of funded CGM in New Zealand and most locations worldwide continues to limit wide-spread and equitable use of CGM, and inadequate glucose data via subsided SMBG remains a major barrier to improving glycaemic outcomes. Alongside these issues are clinician and health-care system mediators of clinical inertia in diabetes management, such as insufficient time and knowledge deficits in non-specialist settings (e.g., primary care) [41].

A strength of our study design is the model of care provision alongside available glucose testing modalities. To our knowledge, this is the first RCT study investigating the use of rtCGM alongside supported specialist care with a focus on participant self-titration of insulin. Previous studies reporting improvements in glycaemic control using CGM have simultaneously observed only modest adjustments to glucose-lowering therapies in both CGM and control groups [25–27, 42]. Participants in these studies accessed care from their usual care providers during the trial phase. Type 2 diabetes is largely managed in primary care in New Zealand, and while heavily subsidised, the costs associated with visiting a primary care clinician remains a barrier to accessing diabetes care [43, 44]. Furthermore, diabetes specialists may be less prone to clinical inertia than primary care clinicians [45], particularly when specialist care adheres to clinical guidelines [46]. Our study design includes equitable, face-to-face and remote access to prescribing DNS for all study participants, with endocrinologist support for DNS as required. Prescribing DNS will assist participants to make changes to their lifestyle and medication regimens to reduce cardiovascular risk. Prescribing DNS will also support participants in both arms of the trial to self-titrate insulin doses between scheduled study review points. This self-titration education will follow an insulin titration guideline (Appendix) to reduce clinician-mediated inertia and bias in regimen changes between study groups. Bias will also be reduced by optimising non-insulin glucose lowering therapies in the run-in phase before participant randomisation.

A further strength of our study design is the continuous and extensive use of rtCGM. Evidence from other studies using CGM in T2D suggests that the length of CGM system use influences glycaemic outcomes. RCT data from studies by Vigersky et al. [26] and Ernhardt et al. [27] showed that participants who used rtCGM for ≥48 days had greater reductions in Hba1c than those who used rtCGM for <48 days (14 mmol/mol/1.3% versus 9 mmol/mol/0.8% [26] and 13 mmol/mol/1.2% versus 7 mmol/mol/0.6% [27]). Our participants will use rtCGM continuously for 12–24 weeks according to randomisation at baseline. As such, with long duration of rtCGM use alongside supported specialist care and participant self-titration of insulin, we expect glycaemic reductions in this group to be greater than previously reported in rtCGM studies of people with T2D.

We also aim to address other deficits in literature including a paucity of qualitative data from indigenous people with T2D using technology, the effect of rtCGM use on dietary and sleep behaviours and cost-benefit analysis of technology use in high-risk indigenous populations with T2D.

The 2GO-CGM trial is likely to afford participants immediate and long-term benefits. We expect that rtCGM use will be well tolerated, and result in a cost-effective increase in TIR, and decrease in hyperglycaemia, without corresponding increase in hypoglycaemia. Our novel, integrated, remote model of care utilising prescribing DNS with endocrinologist support is likely to be effective in improving glycaemic control and cardiovascular risk in both the rtCGM and SMBG groups, as well as offer a transferable solution to real-world clinical care in the face of increasing challenges in workforce shortage and accessibility of care for people with diabetes. As such, we expect the 2GO-CGM study will provide robust evidence-based and scalable solutions for improving care for high-risk Māori and non-Māori with T2D in New Zealand and worldwide.

## Appendix: Insulin titration management algorithm

Sourced from the New Zealand Society for the Study of Diabetes national guidelines on 9th January 2023 available at www.nzssd.org.nz

### For participants on basal insulin only

- Participants in the control group will monitor their glucose levels before meals and bed, and if symptomatic of hypoglycaemia or hyperglycaemia
- If 3 consecutive fasting glucose levels >7 mmol/L then increase dose of basal insulin by 10% or 2 units (i.e. can increase dose every 3 days if monitoring available)
- Stop uptitration of basal insulin if any of the following occurs:
  - Glucose levels <4 mmol/L at any time of day
  - Fasting glucose level < 7 mmol/L
  - Doses of basal insulin reach 0.5 units/kg/day
- At next study visit, start prandial insulin if glucose levels at any time of day consistently >10 mmol/L. The choice of prandial insulin is a shared decision with the participant based on glucose monitoring, participant factors (e.g., timing of meals, variability of diet, daily routine, comfort/ability to inject and monitor glucose levels etc.) and preference. Options include:
- Addition of bolus insulin at largest meal (basal plus)
  - Start rapid-acting insulin at 4 units or 10% of basal dose at largest meal and titrate as below
- Addition of bolus insulin at all main meals (basal bolus)
  - Start rapid-acting insulin at 4 units or 10% of basal dose at all main meals and titrate as below
- Premixed insulin with largest meal (if predominantly one meal per day)
  - Convert daily dose of basal insulin to premixed insulin before largest meal and titrate as below
- Premixed insulin with breakfast and dinner
  - Convert daily dose of basal insulin to premixed insulin with half the dose pre-breakfast and half pre-dinner. Consider a different ratio if one meal larger than the other (e.g., two thirds/one third) and/or the addition of rapid acting insulin at lunch and titrate as below

### For participants on basal plus regimen

- In addition to the above monitoring, participants in the control group should also check their glucose levels two hours after their largest meal (i.e., 2 hours after bolus insulin administered)
- If 2-hour post-meal glucose level > 3 mmol/L than pre-meal glucose level on 3 consecutive occasions then increase dose of rapid acting insulin by 2 units (i.e., can increase dose every 3 days if monitoring available)
- Stop uptitration of rapid acting insulin if any of the following occurs:
  - Glucose levels <4 mmol/L at any time of day
  - Post-meal rise in glucose levels is <3 mmol/L
- At next study visit, add rapid-acting insulin at other meals if glucose levels at other times of day consistently >10 mmol/L
  - Start rapid-acting insulin at 4 units or 10% of basal dose at other meals and titrate as below
  - Consider addition of correction insulin for hyperglycaemia if clinically appropriate

### For participants on basal bolus regimen

- In addition to the above monitoring, participants in the control group should also check their glucose levels two hours after meals (i.e., 2 hours after bolus insulin administered)
- If 2-hour post-meal glucose level is >3 mmol/L than pre-meal glucose level on 3 consecutive occasions then increase dose of rapid acting insulin by 2 units at applicable meal (i.e. can increase dose every 3 days if monitoring available)
- Stop uptitration of rapid acting insulin if any of the following occurs:
  - Glucose levels <4 mmol/L at any time of day
  - Post-meal rise in glucose levels is <3 mmol/L
- At next study visit, if glucose levels at other times of day consistently >10 mmol/L consider:
  - Increasing the dose of basal insulin as per above if glucose levels increase overnight
  - Adding rapid acting insulin with snacks
  - Adding correction insulin for hyerglycaemia

### For participants on once daily premixed insulin

- In addition to the above monitoring, participants in the control group should also check their glucose levels two hours after their largest meal (i.e., 2 hours after premixed insulin administered)
- If 2-hour post-meal glucose level is >3 mmol/L than pre-meal glucose level and fasting glucose level > 7 mmol/L on 3 consecutive occasions then increase dose of premixed insulin by 10% (i.e., can increase dose every 3 days if monitoring available)
- Stop uptitration of rapid acting insulin if any of the following occurs:
  - Glucose levels <4 mmol/L at any time of day
  - Post-meal rise in glucose levels is <3 mmol/L
  - Fasting glucose level < 7 mmol/L
- At next study visit, if glucose levels at other times of day consistently >10 mmol/L consider:
  - Adding bolus insulin at other meals and titrate as above
  - Changing premixed insulin analogue
  - Converting to twice daily premixed insulin and titrate as below

### For participants on twice daily premixed insulin

- In addition to the above monitoring, participants in the control group should also check their glucose levels 2 hours after breakfast and dinner (i.e., 2 hours after premixed insulin administered)
- If 2-hour post-breakfast glucose level is >3 mmol/L than pre-breakfast glucose level and pre-dinner glucose level > 7 mmol/L on 3 consecutive occasions then increase dose of premixed insulin with breakfast by 10% (i.e., can increase dose every 3 days if monitoring available)
- If 2-hour post-dinner glucose level is >3 mmol/L than pre-dinner glucose level and fasting glucose level > 7 mmol/L on 3 consecutive occasions then increase dose of premixed insulin with dinner by 10% (i.e., can increase dose every 3 days if monitoring available)
- Stop uptitration of applicable premixed insulin if any of the following occurs:
  - Glucose levels <4 mmol/L at any time of day
  - Post-meal rise in glucose levels is <3 mmol/L
  - Pre-breakfast or pre-dinner glucose level < 7 mmol/L
- At next study visit, if glucose levels at other times of day consistently >10 mmol/L consider:
  - Adding bolus insulin at other meals and titrate as above
  - Changing premixed insulin analogue (s)

**NB:** Participants will be provided with individualised plans for self-titration of their insulin regimen. Adherence and safety of self-titration will be checked at each study visit. Clinical deviations from the protocol may be required e.g., starting at a lower dose of insulin if concerns over hypoglycaemia. Prandial insulin (either rapid-acting or premixed insulin as appropriate) will be encouraged at all meals where there is a consistent >3 mmol/L increase in glucose levels for 2–4 hours post meals. Non-insulin glucose lowering therapies will be continued throughout the study except for sulfonylureas, which will be stopped if prandial insulin is started at that meal. The initial insulins used in this study will be those prescribed by their normal health care providers and include:

- Basal insulins – glargine (Lantus®) and isophane (NPH; Protaphane®; Humulin® NPH)
- Rapid acting insulins – aspart (NovoRapid®), lispro (Humalog®) and glulisine (Apidra®)
  Premixed insulins – 30% aspart/70% isophane (NovoMix® 30), 25% lispro/75% isophane (Humalog Mix 25®) and 50% lispro/50% isophane (Humalog Mix 50®).
